# Structural Comparison between the Right and Left Atrial Appendages Using Multidetector Computed Tomography

**DOI:** 10.1155/2016/6492183

**Published:** 2016-11-09

**Authors:** Koichi Shinoda, Shogo Hayashi, Daisuke Fukuoka, Ryo Torii, Tsuneo Watanabe, Takashi Nakano

**Affiliations:** ^1^Department of Clinical Laboratory, Gifu University Hospital, 1-1 Yanagido, Gifu-shi, Gifu 501-1194, Japan; ^2^Department of Anatomy, Aichi Medical University, 1-1 Yazakokarimata, Nagakute-shi, Aichi 480-1195, Japan; ^3^Department of Anatomy, Tokyo Medical University, 6-1-1 Shinjuku, Shinjuku-ku, Tokyo 160-8402, Japan; ^4^Research and Development Center for Human Medical Engineering, Gifu University, 1-1 Yanagido, Gifu-shi, Gifu 501-1194, Japan; ^5^Department of Physical Therapy, Chubu Rehabilitation College, 2-2 Wakamiya-cho, Nakamura-ku, Nagoya-shi, Aichi 453-0023, Japan

## Abstract

The three-dimensional (3D) structures of the right atrial appendage (RAA) and left atrial appendage (LAA) were compared to clarify why thrombus formation less frequently occurs in RAA than in LAA. Morphological differences between RAA and LAA of 34 formalin-preserved cadaver hearts were investigated. Molds of RAA and LAA specimens were made and the neck areas, volumes of the atrial appendages (AA), and amount of pectinate muscles (PMs) were analyzed using multidetector computed tomography. In RAA, most PMs were connected to one another and formed a “dendritic” appearance and the inner surface area was smaller than in LAA. RAA had smaller volumes and larger neck areas than LAA. The ratios of the neck area/volume were larger and the amounts of PMs were smaller in RAA than in LAA. The volumes, neck areas, and amount of PMs of RAA were significantly correlated with those of LAA. According to the 3D structure, RAA appears to be suited for a more favorable blood flow, which may explain why the thrombus formation is less common in RAA than in LAA. Examining not only LAA but also RAA by transesophageal echocardiography may be useful in high-risk patients of thrombus formation in LAA because the volume, neck area, and amount of PMs of LAA reflect the shape of RAA.

## 1. Introduction

The atrial appendage (AA) is embryologically derived from the primary atrium and protrudes as an ear-shaped part of the atrium [1]. The inner surfaces of AA are characterized by muscular ridges, that is, the pectinate muscles (PMs). Furthermore, thrombi are frequently formed within AA, which is also the site of atrial natriuretic peptide secretion in mammals [2, 3]. Moreover, AA is considered to modulate the association between atrial pressure and volume. Thus, AA is considered important in the states of atrial volume overload [4, 5].

Clinically, thrombi are more frequently formed in the left atrial appendage (LAA) than in the right atrial appendage (RAA) among patients with atrial fibrillation (AF) [6–13]. Thus, the frequency of thrombus formation is believed to be related to the differences between the luminal surface area and the size of the neck between RAA and LAA [14].

Although a few studies have attempted to compare the structures and functions of RAA and LAA by transesophageal echocardiography (TEE) [4, 6, 7, 14], detailed anatomical differences remain unclear, possibly because the resolution of cardiac ultrasonography is too low to reveal the internal fine structures of AA, for example, PMs. Furthermore, the volume and shape of LAA greatly vary according to the imaging plane [15]. It has been reported that greater bending and spiraling of LAA are associated with a greater variance in images that are obtained from different two-dimensional (2D) planes [16]. Therefore, elucidating the morphological details and accurate observations of AA is difficult by cardiac ultrasound inspection.

Although some studies have described the structure of LAA using molds of gross anatomical structures, none have mentioned RAA. In addition, the usefulness of multidetector computed tomography (MDCT) has been recently established for depicting vascular structures and, thus, is now considered a feasible imaging modality to select a surgical method and for preoperative planning [17]. However, to the best of our knowledge, no previous study has examined the differences in three-dimensional (3D) structures between RAA and LAA. Thus, this study aimed to hybridize these methods (i.e., molds and MDCT images) to analyze morphological and quantitative differences between RAA and LAA.

## 2. Materials and Methods

The hearts of 34 formalin-preserved cadavers (11 males and 23 females; mean age at death, 84 ± 8.4 years), which were donated to the Aichi Medical University, were dissected. The cadavers with a cause of death of embolism or valvular heart disease were excluded.

**Figure 1:**
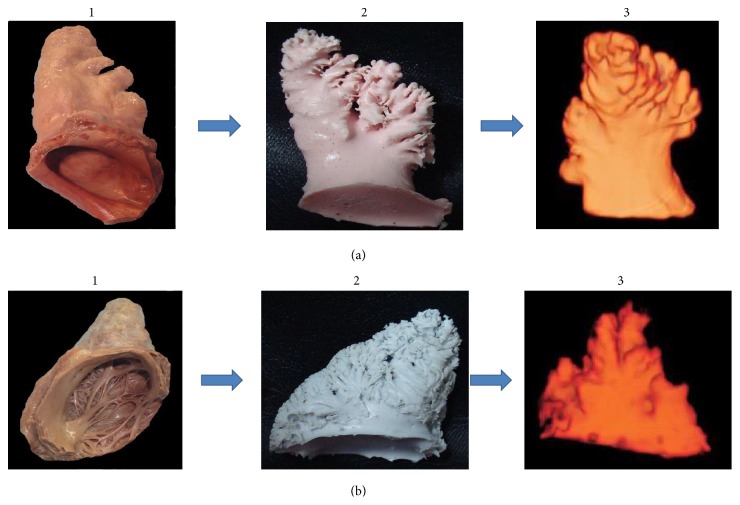
Representative molds of the right atrial appendage (RAA) and the left atrial appendage (LAA). (a)-1 LAA was dissected from the donated body. (a)-2 A silicon mold of LAA. (a)-3 Multidetector computed tomography (MDCT) scanning image of mold of LAA. (b)-1 RAA was dissected from the donated body. (b)-2 A silicon mold of RAA. (b)-3 MDCT scanning image of mold of RAA.

AA was dissected in a manner that preserved some of the atrial wall to identify morphological differences between RAA and LAA, particularly gross anatomical differences in PMs. Then, silicon molds of AA from each heart were produced, which included a portion of the neck between RAA and LAA (silicone acrylic hybrid resin; Nissin Chemical Industry Co., Ltd., Echizen, Japan; mixing ratio of resin to hardener, 100 : 5 w/w; curing time, 7 days). The neck portion of RAA and LAA was defined as the entrance part of the AA for measurement using TEE [7, 18]. The RAA was measured in conjunction with the two parts divided by the tenia sagittalis. Each mold was inspected using MDCT of a Philips Brilliance 64-slice CT system (Royal Philips, Amsterdam, Netherlands). CT scanning protocols: tube voltage, 120 kVp; tube current, 50 mA; detector configuration, 64-row detectors with 0.625 mm section thickness; beam collimation, 40 mm; rotation time, 0.5 s; pitch, 0.515; display field of view, 16 cm; imaging filter, C-types (Figure 1).

### 2.1. Mold Measurements

**Figure 2:**
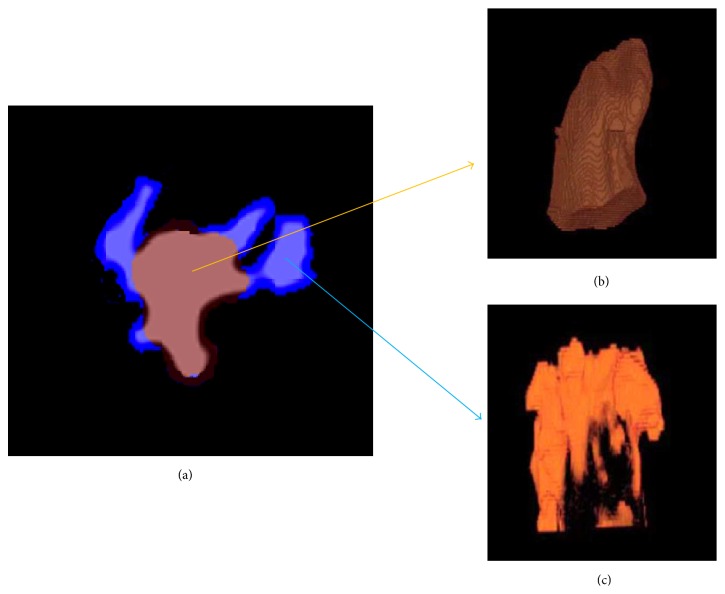
Quantification of the pectinate muscles (PMs) portion of the atrial appendages (AA) by three-dimensional morphological operation. (a) Determination of the PMs portion of the AA by computer analysis (short axis image). (b and c) The PMs were separated from the main body of AA (long axis image). (b) Main body, (c) PMs.

For each mold, the volumes, neck areas, and surface areas of AAs were measured. After binary-coded processing was performed for all volume data in the MDCT image, we performed 3D labeling processing and decided a domain of the whole AA. Computer analysis of overall volume from the number of voxel and neck and surface areas from the number of pixels was performed. From these measurements, the neck area/volume ratio and surface area/volume ratio for each AA were calculated. Furthermore, we excised PMs from each AA by 3D morphological operations (Figure 2) [19] and quantified differences between RAA and LAA. The amount of PMs was determined from the number of voxels using computer analysis.

### 2.2. Statistical Analyses

All measurements are presented as means ± standard deviations. Differences between RAA and LAA were compared using two-tailed paired* t*-tests and Pearson's product moment correlation coefficient. A probability (*p*) value of <0.05 was considered statistically significant.

## 3. Results

### 3.1. Gross Anatomy of RAA and LAA

**Figure 3:**
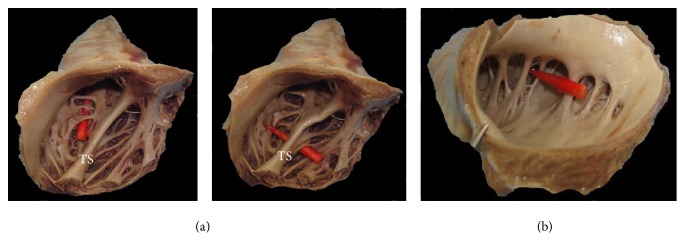
Pectinate muscles (PMs) in right atrial appendage (RAA) and left atrial appendage (LAA). A comparison whether there is intercommunication between PMs. (a)-1,2 PMs in RAA (traffic); (b) PMs in LAA (no traffic). TS: tenia sagittalis.

The inner surfaces of both RAA and LAA are characterized by PMs. There are significant differences between RAA and LAA; as in RAA, most PMs are connected to one another and form a “dendritic” appearance. In contrast, connections between neighboring PMs are less pronounced in LAA (Figure 3).

### 3.2. Neck Areas of RAA and LAA

**Figure 4:**
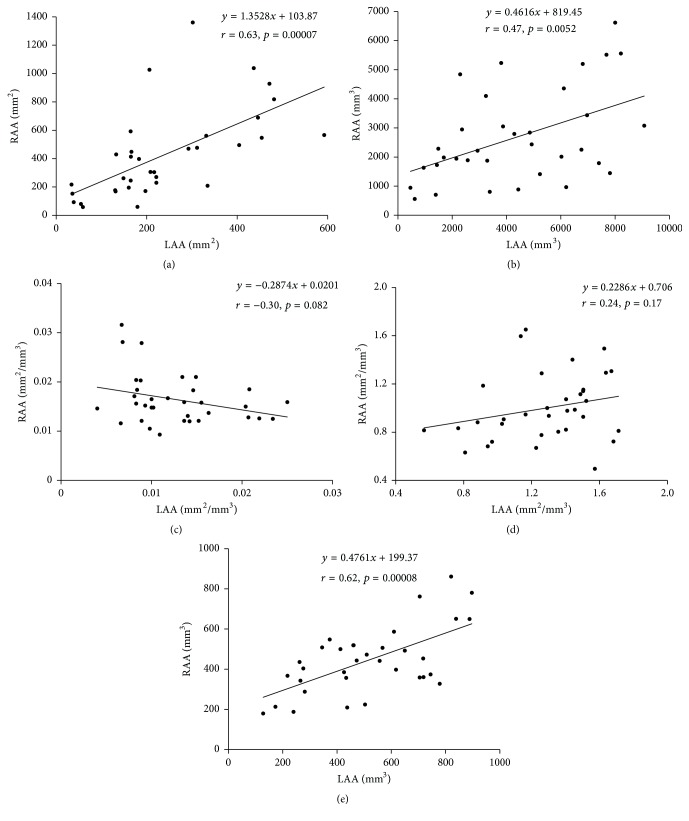
The correlation between the measured values of right atrial appendage (RAA) and left atrial appendage (LAA). (a) Correlation between the neck areas of RAA and LAA. (b) Correlation between the volumes of RAA and LAA. (c) Correlation between the neck area/volume ratios of RAA and LAA. (d) Correlation between the surface area/volume ratios of RAA and LAA. (e) Correlation between the amount of pectinate muscles (PMs) of RAA and LAA.

The mean neck area of LAA was significantly smaller than that of RAA (Table 1). There was a distinct correlation between the neck areas of RAA and LAA (Figure 4(a)).

### 3.3. Volumes of RAA and LAA

The mean volume of LAA was significantly greater than that of RAA (Table 1). There existed a distinct correlation between the volumes of RAA and LAA (Figure 4(b)).

### 3.4. Correlation between Neck Areas and Volumes

**Figure 5:**
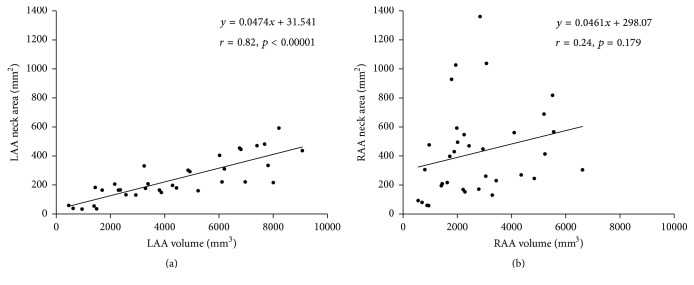
The correlation between the volume and the neck area. (a) Correlation between the volume of left atrial appendage (LAA) and the neck area of LAA. (b) Correlation between the volume of right atrial appendage (RAA) and the neck area of RAA.

There was a significant correlation between the volume of LAA and the neck area of LAA (Figure 5(a)). However, such a significant correlation was not present in RAA (Figure 5(b)).

### 3.5. Neck Area/Volume Ratios of RAA and LAA

The mean neck area/volume ratio of LAA was significantly smaller than that of RAA (Table 1). There was no significant correlation between the neck area/volume ratios of RAA and LAA (Figure 4(c)).

### 3.6. Surface Area/Volume Ratios of RAA and LAA

The mean surface area/volume ratio of LAA was significantly greater than that of RAA (Table 1). There was no significant correlation between the surface area/volume ratios of the RAA and LAA (Figure 4(d)).

### 3.7. Amount of PMs in RAA and LAA

The mean amount of PMs of LAA was significantly greater than that of RAA (Table 1). There existed a distinct correlation between the amounts of PMs of RAA and LAA (Figure 4(e)).

## 4. Discussion

There are few reports on the detailed structures of RAA and LAA, particularly anatomical differences. To the best of our knowledge, this is the first study to compare the morphological characteristics of RAA and LAA.

Regarding the gross anatomy of these structures, LAA is a long, tubular, hooked structure that is usually crenelated with a narrow junction within the venous component of the atrium. In contrast, RAA is broad and triangular with a wide junction. Both RAA and LAA are trabeculated with PMs largely running parallel to each other with a comb-like appearance, although these characteristics are less pronounced in LAA [4]. In fact, in this study, some structural differences in PMs were observed between LAA and RAA (Figure 3). In RAA, most of PMs are connected to one another and form a “dendritic” appearance. In contrast, in LAA, connections between neighboring PMs were less pronounced. These morphological characteristics may participate in stagnation of blood flow and may be regarded as a cause of thrombus formation in LAA.

According to Al-Saady et al. [4], the thrombogenic etiology in AA has not been completely elucidated; however, relative stasis, which occurs in AA owing to its shape and inherent PMs, is considered to play a major role. Yamaji et al. [20] reported that all thrombi are adhered to PMs. The results of this study revealed a lower incidence of PMs in RAA, indicating that the inner surface area of RAA is smaller than that of LAA. However, there was a distinct positive correlation between the amount of PMs in RAA and that in LAA (Figure 4(e)). Moreover, in RAA, there were fewer PMs (Table 1), which are the most frequent sites of thrombus formation, than in LAA, which, in terms of both volume and ruggedness, may explain why thrombus formation in RAA less frequently occurs.

RAA had smaller volumes and larger neck areas than LAA, and thus, the mean neck area/volume ratio of RAA was larger (Table 1). If LAA and RAA had similar size and were homothetic, their mean neck area/volume ratio would be similar. Furthermore, if the composition of AA is constant (homothetic) regardless of it, the neck areas have to correlate to the volumes. Actually in LAA, there was a significant correlation between the volume and neck area (Figure 5(a)). However, in RAA there was no significant correlation between them (Figure 5(b)). These results suggest that the composition of LAA may be less variational but the composition of RAA includes applicable variations. The neck areas (Figure 4(a)), volumes (Figure 4(b)), and amounts of PMs (Figure 4(e)) in RAA were significantly correlated with those in LAA. As for the surface area/volume ratio, which can be considered formally smooth, although RAA had significantly larger neck area/volume ratios and smaller surface area/volume ratios than LAA (Table 1), there was no correlation of these parameters between LAA and RAA (Figures 4(c) and 4(d)).

Atrial thrombus formation may occur in various conditions, such as in AF, in valvular heart disease, in response to a mechanical prosthesis, and in restrictive cardiomyopathy [21, 22]. The present results suggest that thrombus formation within AA may be related to two additional factors, the morphology of AA and the amount of PMs. Atrial thrombi most commonly occur in LAA. According to several clinical studies, RAA thrombi were observed in 0.4%–8.3% of patients with AF, while LAA thrombi were observed in 3.0%–27.3% [6–13] (Table 2). Regarding patients with AF, although both the right atrium and the left atrium are fibrillating, the majority of thrombi are located within LAA, while formation in RAA is uncommon. Moreover, thrombus formation generally occurs in the dilated AA [4, 7, 14, 15, 22–24]. Furthermore, Pollick and Taylor [22] reported that LAA thrombus formation was associated with both poor LAA contraction and dilatation, not only in patients with AF but also in those with sinus rhythm. A study by Veinot et al. [25] revealed that the number of lobulations in LAA was not dependent on age or sex; that is, 54% had at least two lobulations and 80% had two or more. Although this study suggested that there exist great variations in the volume and shape of LAA, the measured size of LAA by 2D-TEE may be affected by the cutting plane [25]. The greater extent of bending and spiraling of LAA is associated with a greater variance in images obtained by different 2D planes. Therefore, this variability should be considered when interpreting images of LAA, particularly when attempting to identify a thrombus [16]. For these reasons, we observed both appendages using 3D reconstruction with MDCT in this study. On the basis of these findings, it is conceivable that, compared with RAA, LAA has structures that promote stagnation of blood flow.

Thrombus formation may occur less often in RAA than in LAA not only because of contractile dysfunction, as evidenced by a stronger spontaneous contrast echo, and decreased flow rate [3, 18], but also because of other causes, such as morphological differences, which we discovered between RAA and LAA. Di Biase et al. [26] classified LAA into four categories based on the morphology (cactus, chicken wing, windsock, and cauliflower types) and reported that patients with non-chicken wing LAA morphology are significantly more likely to have an embolic event. Kimura et al. [27] reported that many patients of embolism have the cauliflower type, which suggests that the more complex the internal structure, the more likely the occurrence of thrombus formation. Because the complexity of the AA reported by Di Biase et al. [26] and Kimura et al. [27] is considered to be related to surface area/volume ratio and amount of PMs, they may have an association with thrombus formation. Furthermore, thrombus formation is more likely to occur in LAA as it has a complex morphology due to the high surface area/volume ratio and amount of PMs (Table 1).

Although much clinical attention has focused only on LAA, Ozer et al. [28] and Sahin et al. [13] reported that RAA thrombus formation might be a source of pulmonary embolism. Therefore, although thrombus formation in RAA is a relatively rare event, when compared with LAA, a thrombus in RAA also has the potential to form an embolism and, thus, should also be considered. In addition, we should keep in mind that it is more difficult to visualize RAA anatomy by TEE compared with that of LAA. The incidence of nonvisualization of RAA anatomy during TEE has been reported to be approximately 1.3%–16% [6, 11]. Moreover, it is next to impossible to visualize the RAA by transthoracic echocardiography, which may also account for the lack of RAA observation. Therefore, we compared the morphological characteristics of RAA and LAA and found significant correlations between the volumes, neck areas, and amounts of PMs. In other words, the structure of LAA formation, which can be comparatively easily investigated by ultrasound, appeared to reflect that of RAA. Although data regarding the formation and occurrence rate of thrombi are limited, observations of not only LAA but also RAA by TEE may be useful in high-risk patients of LAA thrombus formation.

There were several limitations to this study that should be addressed. The first major limitation is the lack of clinical (such as age range and atrial fibrillation) and pathological (such as heart weight, ventricular thickness, chamber dilatation, and previous myocardial infarction) information about the study population. We excluded from the study that the direct cause of death was heart disease and apparent calcification of the mitral valve and/or tricuspid valve. We had no information regarding a past history of heart disease among the cadaver hearts used in this study and, therefore, did not assess coagulation factors related to thrombus formation. Second, absolute values of LAA and RAA measurements were influenced by the fact that all hearts were postmortem specimens, and thus, the unavoidable effect of shrinking as well as the missing intra-atrial pressure, which reduced the diameter and volume of all cardiac structures including atrial appendages, must be taken into consideration. Another limitation is the unavailability of the right and left atrial diameters, which would have provided an insight into the association between the size of the appendages and the size and morphology of the corresponding atria.

## 5. Conclusions

The 3D structures obtained in this study revealed that RAA is suitable for more favorable blood flow, which may be a reason why thrombus formation is less common in RAA than LAA. In contrast, our results also revealed that the volume, neck area, and amount of PMs of LAA reflected the shape of RAA. Hence, TEE of RAA may be useful in patients at high-risk of thrombus formation in LAA. In addition, these results suggested that measuring LAA was important to predict the possibility of future thrombus formation in RAA. To validate our findings, further studies with a larger number of autopsied hearts with and without AF are warranted.

## Figures and Tables

**Table 1:** Measurements of RAA and LAA.

Measurement part		RAA	LAA
Volume (mm^3^)	Mean ± SD	2685 ± 1610	4375 ± 2507
	Range	557–6618	460–9074
	*p* value	*p* < 0.01	

Neck area (mm^2^)	Mean ± SD	425 ± 313	238 ± 145
	Range	59–1361	34–592
	*p* value	*p* < 0.01	

Neck area/volume (mm^2^/mm^3^)	Mean ± SD	0.016 ± 0.005	0.013 ± 0.005
	Range	0.009–0.032	0.004–0.025
	*p* value	*p* = 0.02	

Surface area/ volume (mm^2^/mm^3^)	Mean ± SD	0.999 ± 0.279	1.283 ± 0.295
	Range	0.497–1.651	0.567–1.712
	*p* value	*p* < 0.01	

Amount of pectinate muscles (mm^3^)	Mean ± SD	444 ± 166	514 ± 217
	Range	179–861	128–897
	*p* value	*p* = 0.02	

**Table 2:** Previous reports of the incidence of thrombus formation in the LAA and RAA in patients with AF.

Reference	*N*	RAA & LAA	LAA	% LAA	RAA	% RAA
Manning et al. 1993	94	2	10	11	2	4.3
Manning et al. 1995	230	6	34	15	0	2.6
Klein et al. 1997	53	0	6	11	1	1.9
de Divitiis et al. 1999	90	5	11	12	1	6.7
Rozenberg et al. 2000	230	NA	7	3	1	0.4
Bilge et al. 2000	53	1	13	25	3	7.5
Klein et al. 2001	549	NA	67	12	9	1.6
Sahin et al. 2010	84	NA	23	27	7	8

## Abbreviations

2D: Two-dimensional
3D: Three-dimensional
AA: Atrial appendages
AF: Atrial fibrillation
LAA: Left atrial appendage
MDCT: Multidetector computed tomography
RAA: Right atrial appendage
PMs: Pectinate muscles
TEE: Transesophageal echocardiography.
